# On Puerperal Vaginitis and Laceration as Causes of Vesico - Vaginal Fistula

**Published:** 1879-02

**Authors:** Wm. H. Byford

**Affiliations:** Prof. of Obstetrics, Chicago Medical College


### CHICAGO MEDICAL JOURNAL & EXAMINER.

VOL. XXXVIII.—FEBRUARY, 1879.—NO. 2.

[In these pages dimension and weight are expressed in terms of the Metric System, and temperature in degrees of the Centigrade Scale.]

### Original Lectures.

### ARTICLE I.

ON PUERPERAL VAGINITIS AND LACERATION AS CAUSES OF VESICO-VAGINAL FISTULA. Clinical lecture by WM. H. BYFORD, M.D., Prof. of Obstetrics, Chicago Medical College.

Gentlemen: The main object I have in view in my clinical lecture to-day, is to develop more fully than has yet been done—so far as I am informed—the *modus in quo* of the formation of vesico-vaginal fistula from prolonged pressure of the fœtal head in passing through the pelvis. You have no doubt been surprised at the great damage to the soft parts in one case we have in the Mercy Hospital, resulting from ignorance and negligence in the management of, or rather mismanagement of, her labor; and without a somewhat detailed account of the morbid processes following the confinement, you would hardly be able to comprehend with any clearness how they were effected.

I will relate in a concise manner the history of the labor so far as we were able to get it, and again describe to you the condition of the pelvic viscera when we made the examination. I think the explanation I intend to give you will be more interesting and instructive, if I add a short account of the case upon which you saw me operate yesterday. The resulting injury was very much less in this than in the other case, but it was brought about by the same cause—an impacted head—and the subsequent vaginal inflammation was the same in character; differing however very greatly in degree. I will conclude the lecture by a third case recently operated on in your presence, in which the accident originated in a very different manner, and call your attention to the widely different conditions of the vaginal cavity, and the appearance of the fistula itself. If I am successful in making myself understood I think I will prove to you that the effect of the after mismanagement of these disastrous cases of midwifery was as pernicious as that of the labor itself, and that much of the resulting damage might have been avoided by judicious subsequent treatment.

Our first case is Mrs. W—— from western Iowa. This patient, gentlemen, is a primipara, twenty-five years of age. As you are aware, she is now in the hospital the second time. Last April she first entered the institution in a very enfeebled condition; three months after her confinement. She then was suffering great pain in the pelvic regions, of a dull aching character, with a sense of great heat where the vagina ought to be, her pulse was steadily over one hundred strokes to the minute, and her temperature from one to two degrees above the normal standard; she had a very poor appetite, and was under the necessity of taking some form of opium to secure rest at night. She was greatly emaciated, and prostrated to such a degree that she was unable to sit up more than a few minutes at a time. Her extremities were cold and bathed a good share of the time in a very liquid perspiration. When we came to examine the vagina, we found it to be the receptacle of urine and fœces; the whole of both of these excretions passed through the canal, which was large and very irregular in shape, bearing no resemblance to the normal vagina. Upon separating the labia, we found about three quarters of an inch of the urethra left, the internal portion of it entirely gone; no trace of vaginal mucous membrane could be found, except a small patch just above the perineum on the posterior wall. Above this the recto-vaginal septum was removed for two inches, and for this distance the posterior part of the great irregular cavity was formed by the mucous membrane of the rectum; at the upper part of the recto-vaginal opening was a dense semilunar cicatricial band sweeping half way around the pelvis, the left hand extremity of which terminated lower down than the right. The anterior and much of the lateral walls of the cavity were formed by the mucous lining of the upper wall of the bladder, so much of the substance of the bladder was gone that there was not enough left to collapse into this cloaca. The perineum was very deficient, measuring only about half an inch antero-posteriorly, but so much of the external sphincter was left as to contract the anus very firmly; several large cicatricial deposits bound the sides of this great cavity to the pelvic bones. The external organs were inflamed and excoriated so as to add very much to the suffering of the patient. As you see her to-day, after a lapse of six months, she is very much improved in general health, she has the color, flesh and vivacity of good health; and comes to us, hoping to have an operation performed that will restore the integrity of the vagina, and is very much disappointed because she is told that the time has not come to make any surgical effort to improve her condition. We find however that nature has made a wonderful improvement in her local condition.

I forgot to say to you that in the first examination I entirely failed to find the os uteri.

The whole vaginal cavity is very much contracted and the cicatricial bands more prominent than at the first examination, and the opening into the rectum has decreased in size to such an extent as to prevent the fæces, when hard, from passing through it, and now she actually evacuates a portion of the contents of the rectum through the anus. Now what is the best we may hope for by an operation in the future? It is not unreasonable to suppose that, after a while, we may be able to close the recto-vaginal fistula, and then by closing the vulva, enable her to pass the urine through the urethra. This is all I think we shall ever be able to do for her. But this will very much improve her condition. I cannot yet find the os uteri, although I can feel the body of the uterus through the rectal opening. She has not menstruated, and it is probable she never may. Possibly the uterine cavity is not obliterated, and it may communicate through some very small undiscovered canal, with the vagina, or the vesical cavity may be so completely obliterated as to cause the menses to be retained. Let us look at the obstetrical history of this case. It is very imperfect, but yet sufficient is revealed to enable us to get at the main facts. She was confined in the latter part of January, 1877—she was in labor three and a half days, during the last two of which the head of the fœtus rested in the pelvic cavity. She was at last delivered by the perforator and crotchet. She was first attended by a midwife, who for three days held out the hope to the patient and friends that nature would deliver the woman "all right." At the end of this time a physician was called in, but having no instruments a further delay was necessary before a second could be procured with the requisite means of terminating this disastrous labor. The child of course was dead, and in a state of decomposition when delivered; the patient was in a state of great exhaustion, and for several weeks her life was jeapordized by what was called puerperal fever. On the fourth day the discharge from the vagina became very fetid, and masses of flesh, as the woman expresses it, passed or were removed from the vagina by the attendants, and on the sixth day the urine and fæces commenced to pass the vaginal orifice. It was a month before hopes of her recovery were indulged in, and she was barely able to be brought from her home in a palace-car to Chicago, in three months from time of confinement.

Before making any comments on this case let us review the history of the second case, that of Mrs. M——, from Michigan, whom we operated upon yesterday for vesico-vaginal fistula. She is also a primipara, thirty years of age, she is a very large fleshy woman, weighing over two hundred pounds. She was confined in January, 1878. Her labor is described by her and her friends as very severe and protracted. From her statement there can be no doubt but that the head remained immovable in the pelvis for twenty hours, when it was removed by the forceps. She was in care of a midwife, who assured her from time to time, that the child was about to be born, as it could be seen when the external parts were pressed open. This declaration enabled the midwife to postpone the call for aid, until, as before stated, the head had been in that position twenty hours or more. At the time she was delivered the patient was very much exhausted, had vomited a number of times, her extremities were cold, pulse rapid and her mind wandering ; in ten or twelve hours re-action had taken place, the temperature was increased, the pulse remained frequent, the external parts were swollen and there was tenderness and tumefaction in the lower part of the abdomen. I could not learn that the urine was evacuated by the catheter at any time, but the patient assured me that she passed it in small quantities through the urethra. For two weeks she had what was called a mild form of child-bed fever. During this time there were vaginal discharges that became very offensive, and in something over a week the urine commenced passing involuntarily ; at first, it was supposed, through the urethra, but finally there could be no doubt that it all came by way of the vagina. Although we cannot arrive at definiteness, I think we are justified in saying that as early as the eighth day the fistula between the bladder and vagina was established.

When she came here an examination revealed a fistula 4 Cm. in the long diameter and about $2\frac{1}{2}$ Cm. in the shorter. The upper end of the opening extended to the left of and involved the cervix about 1 Cm. The lower extremity reached across the median line of the vesico-vaginal septum, and extended down to the urethra. This description, as you see, makes the direction of the fistula oblique to the general direction of the vagina and ovoid in shape. This fistulous opening must have resulted from a considerable loss of tissue by sloughing or ulceration or, more probably, both. Fortunately there were no cicatricial bands, the uterus was quite movable, and the vagina supple and otherwise healthy.

This description of these two cases is given with a view to illustrate the usual manner of their occurrence. You can hardly doubt, from a review of the circumstances attending the labors, that protracted pressure of the fœtal head upon the soft parts nipped between the solid bones of the skull and the bony pelvic wall, was the cause of the vesico-vaginal fistula. This cause is recognized by most writers upon the subject, and thus far their explanation and testimony as to the occurrence of the accident is clear and to the point. The general expression of the manner in which this pressure acts, is so loose and uncertain, however, as to mislead the student, both in his understanding of the process through which the damage takes place and the proper method of treating the consequences. The idea conveyed is that the pressure of the head produces sloughing, and that the fistula is left as the result of the loss thus arising. We are led to infer that this loss of tissue is the immediate effect of the pressure, that death of the portion of the vaginal septum takes place at the time of the pressure, and consequently the damage is both immediate and irremediable. That embarrassment of the capillary circulation in some of the more aggravated cases may be so great, as to make reparation impossible, I can conceive; but from somewhat extensive observation I am convinced that these cases are exceptional, and that by an intelligent understanding of the subject, the sloughing might be avoided in a large number of cases, and in all greatly limited in extent; indeed I believe that sloughing in the proper sense of the term is not generally the cause of the fistula, but that it is more frequently the effect of extensive ulceration.

One of the best authorities on the subject of vesico-vaginal fistula, Dr. Emmet, of New York, read an elaborate paper at the recent meeting of the American Gynæcological Society, in which he tabulated the conditions under which it occurs. The only point made in that paper to which I desire to call your attention, is that the *average* time in the 161 cases therein mentioned, when the urine made its appearance in the vagina, was nine days after delivery. In some cases the time was, of course, much longer; in others, much less than this average time; but in none did that excretion find its way through the septum immediately after the birth of the child.

The question naturally arises, in this connection, what was transpiring in the vagina from the time of delivery up to the period of the completion of the fistulous opening and the appearance of the urine in the vagina? Can it be that one, two and sometimes three weeks are required for the separation of a slough formed at the time of delivery? I think not, and again say that I

believe immediate death to the parts involved is the rare exception.

Then how is the fistula usually produced?

I answer, by the ulceration attending the severe inflammation caused by the detention of the head. Sometimes, doubtless, the ulceration is preceded by sloughing, but not always, and when thus preceded by the sloughing of a part of the thickness of the vaginal wall, the ulceration destroys the remaining substance at that point.

For twenty years I have been in the habit of describing in a distinct manner to my classes what I call puerperal vaginitis. In my work on the "Medical and Surgical Treatment of the Diseases and Accidents Peculiar to Women," the first edition of which was published in 1865, I have devoted a section to the description of this affection. You will there find the causes, symptoms and mode of treatment given with a view to avoid the disastrous consequences that usually result from the disorder, and I am convinced that if the teaching there given were properly followed out, that vesico-vaginal fistula would often be avoided, even after the protracted retention of the head had initiated the processes which, if not arrested, would eventuate in that deplorable lesion.

As you have seen by the imperfect history of the two sample cases I have detailed, the subjects of these badly conducted labors are left in a state of great exhaustion, which is followed by a rapid pulse, elevation of temperature and other symptoms of a low grade of inflammatory fever, the severity of which is indicative of the amount of mischief going on in the vagina and neighboring viscera. Locally there is tumefaction of the lower portion of the abdomen, with tenderness on pressure; the labia and perineum are swollen and very sensitive, and the whole vaginal cavity is hot, dry and tumefied; the urethra is inflamed and very tender to the touch, and either from this condition or partial paralysis of the bladder, or both, the urine is retained. In a short time the discharges from the vagina become muco-purulent, a little later fœtid, and before long shreds of decomposed mucous membrane and fibrous tissue escape, and eventually urine makes its appearance among them. A careful examination of the vaginal walls with the finger, on the second day and thereafter, will detect patches more tumefied and roughened, pointing out the localities where the walls are on the point of yielding to the processes of ulceration and sloughing.

In some instances, no doubt, these ulcerating points mark the boundaries of necrosed patches of the vaginal wall, and in rare cases a slough does separate the whole thickness of the septum; but in the majority of them the slough, when it drops from its position, only partially perforates the vesico-vaginal septum, while ulceration completes the damage. Frequently, however, especially in the less grave forms of injury, ulceration does all that is required to lay open the division between the vagina and bladder. In the former and more intense forms of injury, the perforation will take place in four or five days; in the latter, less mischief is done, and the urine does not flow from the vagina until one, two and in some instances three weeks have elapsed. I am sure you will agree with me, gentlemen, that in this time there is ample opportunity to palliate or lessen the consequence of the damage done by the pressure of the fœtal head in all cases, and in some to completely save the unfortunate patient from the disastrous condition presented in our second case. As I am not now dealing with the obstetrical aspect of this question, I do not think it necessary to speak to you of the management of labor as a preventive measure. You will be taught that branch of the subject by my very able associate, in a manner that will enable you to avoid all difficulties of the kind when you have exclusive management of cases of impacted head.

Before detailing the treatment necessary to be followed after these neglected cases of labor have terminated, I desire in a more emphatic manner to call your attention to the condition of the bladder as one of great importance in effecting subsequent mischief. It may be incapable of expelling its contents, so that it becomes distended, and the vesico-vaginal septum stretched to such an extent as to encourage and perhaps complete the breach of continuity; the sloughing and ulceration have begun by simple traction on the weakened points. But this is not all; the capillary circulation in the distended part is so embarrassed as to promote ulceration, while the weight of urine on the septum is another item of mischief.

Of the treatment then I should say, that to maintain an empty condition of the bladder is the first requisite, and is indispensable to success. The sensitiveness caused by the inflammation will sometimes render this a difficult task. In most instances, however, the bladder will tolerate one of Dr. Sim's catheters. This instrument, armed with an elastic tube long enough to carry the urine into a receptacle by the side of the bed, will keep the bladder empty, at the same time that it prevents that secretion from coming in contact with the inflamed labia. The stationary catheter will cause less irritation than the frequent introduction of one for the same purpose. Another thing quite important is to prevent distension of the rectum by fæces and gas, by first washing that organ out thoroughly, so as to free it from all solid contents, and then placing an elastic tube in it to permit the escape of flatus. The vaginal inflammation should be treated for the first day or two by large injections of hot water from four to six times a day, for the purpose of stimulating the capillary circulation, and keeping the vagina clean. When shreds of membrane begin to make their appearance or the discharges to contain pus, it will be necessary to examine the vaginal walls with the finger to ascertain the locality of the slough or ulcer. On the anterior wall just behind the symphysis, and perhaps a little to the left, the roughened, uneven surface will indicate the position, size and depth of the mischief going on. To these places special attention should be directed ; applications should be made to them with a view of arresting the ulcerative process. By means of a flexible tube or a Lente's ointment carrier, directed by the finger, we may make almost any desired application ; one of the best I think is a solution of sulphate of copper of the strength of ten grains to the ounce of water. This can be thrown upon the parts through a tube in quantities just sufficient to saturate the surface once a day. A solution of nitrate of silver of about the same strength will also answer a good purpose. These local applications will not enable us to dispense with the use of the large hot water injections ; these must be kept up steadily. I have no hesitancy in believing that I have on several occasions prevented the perforation of the bladder by this course of treatment.

We should by no means neglect general treatment in these important cases. The internal administration of quinia and iron, and liberal nourishment are all-important to improve the reparative powers of the system ; while the judicious use of opium will relieve the pain and suffering which are sometimes very great. The treatment should be persevered in until the ulceration is entirely healed.

Before closing my lecture, gentlemen, I wish to call your attention to another case which I presented to you a few days since, as illustrative of another much rarer course of vesico-vaginal fistula, viz : that of Mrs. M——, 28 years of age, from the interior of this State. She is a small woman, muscular and of very vigorous form. She was confined with her fourth child, five months before entering the hospital. Her three previous labors have been natural and of quite short duration. The last labor terminated in about four hours from the time of commencement ; her physician did not arrive until a few minutes before the expulsion of the head. When he made an examination he found the bladder prolapsed, and pressed very forcibly out between the labia. He first thought the protrusion was the bag of water, but before he had time to verify his diagnosis there was a gush of fluid and the head was delivered. The rest of the fœtus was almost immediately expelled, and was soon followed by the membranes and placenta.

At this time the true state of things was not suspected, but the continued dribbling of urine led to a digital examination and discovery of the fistula. From this statement of the case we cannot doubt but that the distended and prolapsed bladder was torn by the force of uterine contractions. I have never before known this accident to occur; whether there are such cases on record I am not prepared to say.

At the time of the operation, I drew your attention to the fact that while this fistula extended from the cervix uteri to the urethra, it was in the form of a fissure. There was no loss of substance as is always the case in fistula caused by puerperal vaginitis.

